# IRF3 Negatively Regulates Toll-Like Receptor-Mediated NF-κB Signaling by Targeting TRIF for Degradation in Teleost Fish

**DOI:** 10.3389/fimmu.2018.00867

**Published:** 2018-04-26

**Authors:** Xueyan Zhao, Ruixuan Huo, Xiaolong Yan, Tianjun Xu

**Affiliations:** ^1^Key Laboratory of Exploration and Utilization of Aquatic Genetic Resources, Shanghai Ocean University, Ministry of Education, Shanghai, China; ^2^College of Marine Science, Zhejiang Ocean University, Zhoushan, China; ^3^International Research Center for Marine Biosciences at Shanghai Ocean University, Ministry of Science and Technology, Shanghai, China; ^4^National Pathogen Collection Center for Aquatic Animals, Shanghai Ocean University, Shanghai, China

**Keywords:** IRF3, TRIF, NF-κB, negative regulation, ubiquitination

## Abstract

NF-κB signaling is tightly regulated and essential to innate and adaptive immune responses, its regulatory mechanism remains unclear in various organisms, especially teleosts. In this study, we reported that IRF3 can negatively regulate TRIF-mediated NF-κB signaling pathway. Overexpression of IRF3 can inhibit TRIF-mediated NF-κB signaling pathway. However, knockdown of IRF3 had an opposite effect. IRF3 can promote the degradation of TRIF protein in mammal and fish cells, but this effect could be inhibited by MG132 treatment. Furthermore, we found that the inhibitory effect of IRF3 primary depended on its IRF association domain domain. IRF3 is crucial for the polyubiquitination and proteasomal degradation of TRIF. Our findings indicate that IRF3 negatively regulates TLR-mediated NF-κB signaling pathway by targeting TRIF for ubiquitination and degradation. This study provides a novel evidence on the negative regulation of innate immune signaling pathways in teleost fish and thus might provide new insights into the regulatory mechanisms in mammals.

## Introduction

Vertebrates have evolved various immune defense systems to protect themselves against invading microorganisms and eliminate infective pathogens (1). Innate and acquired immunity composed of two branches of the immune system. The innate immune system is the first line of host defense against pathogens, such as viruses and bacteria, and can recognize a limited, but highly conserved set of molecular structures known as pathogen-associated molecular patterns (PAMPs) (1). PAMPs are recognized by several classes of pattern-recognition receptors, such as toll-like receptors (TLRs), RIG-I-like receptors (RLRs), NOD-like receptors and C-type lectin-like receptors (2–5). The specific ligands are recognized by different receptors, and the ligand–receptor binding results in the activation of common downstream pathways, such as NF-κB, MAPK, and type I interferon, which further induce the expression of cytokines and chemokine genes that facilitate the clearing of pathogens (6). However, aberrant immune responses occur, which leads to severe or even fatal bacterial sepsis, autoimmune, and chronic inflammatory diseases (7, 8). Therefore, these crucial signaling pathways have to be tightly regulated to maintain immune balance, which is essential to both innate and adaptive immunity.

Host cells can recognize viral products through PRRs, especially TLRs and RLRs, on the surfaces of the cytomembranes or in the cytoplasm during virus infection, and then the downstream IFNs or other relative cytokine production processes are triggerred through signal transduction, thereby establishing the antiviral state of a host (9–11). However, the excessive immune response often leads to many inflammatory and autoimmune diseases. Thus, a variety of regulatory factors are needed to tightly regulate the TLR or RLR signaling pathway to maintain immune balance. For example, NLRX1 inhibits the activity of RLH and interferon-β promoter, which is mediated by MAVS (12). NLRX1 can inhibit TLR-induced NF-κB signaling by interacting with TRAF6 or binding directly to an IKK complex; it also inhibits the phosphorylation of IKK, further facilitating the inhibition of NF-κB activation and releasing of the proinflammatory cytokines (6). The COX5B physically interacts with MAVS and negatively regulates the MAVS-mediated antiviral pathway (13). In addition, the ubiquitin E3 ligase RAUL can negatively regulate type I interferon through the ubiquitination of transcription factors, such as IRF7 and IRF3 (14).

Toll-like receptors are type I transmembrane proteins and contain extracellular ectodomains with leucine-rich repeats, which are transmembrane domain; and an intracellular toll interleukin (IL)-1 receptor domain for the recruitment of downstream adapter proteins. To date, five of adaptor proteins have been identified, including MyD88, Myd88 adaptor-like, TRIF, TRAM, and SARM (15–17). Thus, the signaling pathways are divided into the MyD88-dependent and TRIF-dependent pathway. Recruited MyD88 proteins promote the phosphorylation of IRAKs and subsequently activate the expression of TRAF6, then the NF-κB and MAPK pathways are activated and proinflammatory cytokines are induced (18–20). TLR3 and TLR4 can recruit of TRIF and subsequently to activate the TRIF-dependent pathway, then TRIF interacts with TBK1 to activate downstream IRF3 and NF-κB, consequently TLR3 and TLR4 inducing signaling pathway can induce the producing of type I IFN and proinflammatory cytokines (18–20). To avoid harmful and inappropriate in inflammatory responses, a variety of mechanisms can negatively regulate the TLR signaling pathways (16). Furthermore, a variety of cases that regulated TRIF-mediated signaling pathway have been found. For instance, ADAM15 can negatively regulate TRIF-mediated NF-κB and IFNβ reporter gene activity (21). TRIM38 can negatively regulate the TLR3-mediated type I interferon signaling pathway by targeting TRIF for degradation (22). WWP2, an E3 ligase, can negatively regulate TLR3-mediated innate immune response by targeting TRIF for ubiquitination and degradation (23). To date, many instances that IRFs regulate MyD88-mediated signaling have been confirmed. Such as, IRF4, which competes with IRF5 for interacting with MyD88, acts as a negative regulator of TLR signaling (24). IRF5 interacts with MyD88 and TRAF6 and then activates cytokine gene transcription (25). However, there are very few reports that IRF family member can regulate TRIF-mediated NF-κB pathway signaling.

Although many genes in mammals are involved in the regulation of TLR signaling pathways, the mechanisms involved the regulation of these pathways are rarely reported in fish. Miiuy croaker (*Miichthys miiuy*) is an economically important marine fish. The study of this species have been conducted in-depth from transcriptome and whole-genome (26), to immune genes (27, 28), which left miiuy croaker as an excellent model for studying the mechanisms of some molecules in regulation of immune response of fish (29–31). In this study, we confirmed that IRF3 negatively regulated TRIF-mediated NF-κB signaling pathway by targeting TRIF for degradation in teleosts. Overexpression of IRF3 can inhibit TRIF-mediated NF-κB signaling pathway, whereas knockdown of IRF3 has an opposite effect. Fundamentally, IRF3 promotes TRIF degradation *via* the ubiquitin–proteasome pathway. In addition, we found that the IRF association domain (IAD) of IRF3 is crucial for TRIF proteasomal degradation. Furthermore, we found that IRF3 may inhibit TRIF expression and consequently result in decreasing the expression of downstream inflammatory cytokines. This study not only provides an evidence for the regulatory mechanism of TRIF signaling by IRFs and enriches the content of TLR signaling pathway in teleosts, but also might provide new insights into the regulatory mechanism in mammals.

## Materials and Methods

### Plasmids Construction

To construct the expression plasmids, TRIF of the miiuy croaker was cloned into the *Kpn* I and *BamH* I sites of pEGFP-N1 and to the *Kpn* I and *EcoR* I sites of pCDNA3.1 (Invitrogen) with Flag tag; IRF3 of the miiuy croaker was cloned into the *Kpn* I and *Xba* I sites of pCDNA3.1 with myc tag; TBK1 of the miiuy croaker was cloned into the *Hind* III and *EcoR* I sites of pCDNA3.1 with HA tag; STAT1a of the miiuy croaker was cloned into the *BamH* I and *EcoR* I sites of pCDNA3.1 with myc tag; p65 of the miiuy croaker was cloned into the *Hind* III and *Kpn* I sites of pCDNA3.1 with Flag tag. The TAK1 expression plasmids were conducted using a ClonExpress II One Step Cloning Kit (Vazyme) with specific primers then was cloned into the pcDNA3.1. And the series of deletion mutants of IRF3, including IRF3ΔDNA binding domain (ΔDBD), IRF3ΔIAD (ΔIAD), and IRF3ΔSRD (ΔSRD), were generated by PCR based on the IRF3 recombinant plasmid by using specific primers. The pRK5-HA-Ubiquitin-WT (Ubiquitin-HA) plasmid was purchased from Addgene. And the oligonucleotide of IRF3-shRNAs were designed and ligated into *BamH* I and *EcoR* I of pSIREN-RetroQZsGreen1 vector (Clontech). The recombinant plasmid was confirmed by DNA sequencing. All primers were listed in Table 1. All of the plasmids were extracted using EndotoxinFree Plasmid DNA Miniprep Kit (Tiangen).

**Table 1:** PCR primer sequence information in this study.

Primers	Sequences (5′–3′)
**Vector construction**	

TRIF-KpnI-F	CGGGGTACC ATGAGCCGCGAGGGAGAA
TRIF-EcoRI-R	CCGGAATTCCTAAAGACATTGCTCATC
TRIF-GFP-KpnI-F	CGGGGTACCATGAGCCGCGAGGGAGAAG
TRIF-GFP-BamHI-R	CGCGGATCCCTAAAGACATTGCTCATCTG
IRF3-KpnI-F	CGGGGTACCATGTCTCATTCTAAACCTCTGCTCATC
IRF3-XbaI-R	TGCTCTAGAGTGTCAGTACAGCTCCATCATCTC
IRF3-shRNA1-F	GATCCGCTTCAAACTGGTCTCTGATTCAAGAGATCAGAGACCAGTTTGAAGCTTTTTTG
IRF3-shRNA1-R	AATTCAAAAAAGCTTCAAACTGGTCTCTGATCTCTTGAATCAGAGACCAGTTTGAAGCG
IRF3-shRNA2-F	GATCCGACTGAAGAGCTGACTCAATTCAAGAGATTGAGTCAGCTCTTCAGTCTTTTTTG
IRF3-shRNA2-R	AATTCAAAAAAGACTGAAGAGCTGACTCAATCTCTTGAATTGAGTCAGCTCTTCAGTCG
IRF3-shRNA3-F	GATCCGGATAACATACCTGCCTTCTTCAAGAGAGAAGGCAGGTATGTTATCCTTTTTTG
IRF3-shRNA3-R	AATTCAAAAAAGGATAACATACCTGCCTTCTCTCTTGAAGAAGGCAGGTATGTTATCCG
IRF3-dDBD-EcoRI-F	CCGGAATTCAACTCTAGTGCTGGATCC
IRF3-dDBD-EcoRI-R	CCGGAATTCAGCGTAATCTGGAACATCGT
IRF3-dIAD-EcoRI-F	CCGGAATTCCCAGACAACAGGCCTTGGGAG
IRF3-dIAD-EcoRI-R	CCGGAATTCATCTCCATCTCTGGTCTTGTT
IRF3-dSRD-EcoRI-F	CCGGAATTCCTCGAAGAGATGATGGAGCTG
IRF3-dSRD-EcoRI-R	CCGGAATTCGGCGCCGCCTCCAACAGCCA
TAK1-BamHI-F	CTTGGTACCGAGCTCGGATCCATGTCTCTAACGTTACCGTCCGC
TAK1-EcoRI-R	TGATGGATATCTGCAGAATTCGACATGCAGGACACAGTAGAATGC
TBK1-HindIII-F	CCCAAGCTTATGCAGAGCACCACCAACT
TBK1-EcoRI-R	CCGGAATTCCCGTTTGTTCACGAACTCA
P65-HindIII-F	CCCAAGCTTATGGCGGATGTGTACGGAT
P65-KpnI-R	CGGGGTACCCTGAAGGCTAAAGGAGCAG
STAT1a-BamHI-F	CGCGGATCCGCGCAGTGGTGCCAGCTC
STAT1a-EcoRI-R	CCGGAATTCTCAGTTTTGGTCTGGAAACTC

**Real-time PCR**	

IRF2-RT-F	CGAGGAGGTGAAGGATAAA
IRF2-RT-R	GGATGCCTGAGATGCTGT
IRF3-RT-F	GAATGATGCTGCTAACCC
IRF3-RT-R	CGACTGGAGTCTCAAACG
IRF6-RT-F	AGAAATTGGCGAGGAAGA
IRF6-RT-R	ACAGGGCGTCAGGTAGAG
RIP1-RT-F	GTCAAGTTGCTGGGTGTAA
RIP1-RT-R	TCTATGATGATTCTGCCTTT
TRAF3-RT-F	GAGGTGCCGTGTCCGTTGGGTAA
TRAF3-RT-R	TCGCCATCATTCTCAGGTGTTCAGC
TRAF6-RT-F	ATGATGGAAAAGGAACGGGAAT
TRAF6-RT-R	TCGGACAGCGAACAGTTAGTGA
TNFα-RT-F	GTTTGCTTGGTACTGGAATGG
TNFα-RT-R	TGTGGGATGATGATCTGGTTG
IL8-RT-F	AGCAGCAGAGTCTTCGT
IL8-RT-R	TCTTCGCAGTGGGAGTT
IL1β-RT-F	CATAAGGATGGGGACAACGAG
IL1β-RT-R	TAGGGGACGGACACAAGGGTA
β-actin-RT-F	GAGCCGCACGCTTCTTT
β-actin-RT-R	CTGCTGTAGCCGAGGAC

### Cell Culture and Transient Transfections

Miiuy croaker macrophages were aseptically isolated from the head kidney as previously reported (32), and cultured in l-15 medium (Hyclone) supplemented with 20% FBS (Fetal Bovine Serum, Gibco, Lot 1861242) in 4% CO_2_ at 26°C. Miiuy croaker kidney cell lines (MKC) were cultured in l-15 medium supplemented with 15% FBS in 4% CO_2_ at 26°C. The Miiuy croaker macrophages and MKC cells of miiuy croaker were transfected with siRNAs or plasmids using Lipofectamin 3000™ (Invitrogen). HEK293 cells were cultured in DMEM medium which contained the 10% FBS, 2 mM l-glutamine, 100 U/ml penicillin, and 100 mg/ml streptomycin, and under humidified conditions with 5% CO_2_ at 37°C. Fish EPC cells were cultured in medium 199 which contained the 10% FBS, 2 mM l-glutamine, 100 U/ml penicillin, and 100 mg/ml streptomycin, and under humidified conditions with 5% CO_2_ at 28°C. HEK293 and EPC cells were transfected with various plasmids by using Lipofectamin 2000™ (Invitrogen) and X-tremeGENE HP (Roche), respectively. In addition, the proteasome inhibitor (MG132) or cycloheximide (CHX) was added into medium at 36 h post-transfection.

### RNA Isolation and qRT-PCR Analysis

Total RNA was isolated from miiuy croaker macrophage cells with TRIzol reagent (Takara) according to the manufacturer’s instructions. The cDNA was synthesized by using the FastQuant RT Kit (Tiangen) including DNase treatment of RNA to eliminate genomic contamination. qRT-PCR was performed with a SYBR Premix Ex Taq kit (TaKaRa) on the 7500 system (Applied Biosystems, USA). PCR cycling conditions were as follows: 10 s at 95°C, and followed by 40 cycles consisting of 5 s at 95°C, then 31 s at 60°C. All primers used for qRT-PCRs are shown in Table 1 and β-actin was used as an internal control.

### Luciferase Reporter Assays

HEK293 cells were transfected with expression plasmids and NF-κB, IFNβ or ISRE reporter gene plasmids, and Renilla luciferase reporter plasmid (pRL-TK) was used as the internal control. The proportion of the amount of plasmids: pRL-TK: NF-κB, IFNβ, IFNr, or ISRE reporter gene plasmids are 1:10. Reporter luciferase activities were measured by using the dual luciferase reporter assay system (Promega). The control group used the equal amount of corresponding empty vector compared with the experimental group. For each experiment, the results were done in triplicate for each experiment, and three independent experiments were conducted.

### Prokaryotic Expression and Polyclonal Antiserum

The full-length CDS region of miiuy croaker TRIF was cloned into *EcoR* I/*Xho* I sites of pGEX-4T-1 vector (GE) to construct pGEX-4T-1-TRIF plasmid. Then, the plasmid pGEX-4T-1-TRIF was transformed into the BL21 (DE3) *Escherichia coli* strain and expressed as a protein containing TRIF fused with GST. The fusion protein was induced by isopropyl β-d-thiogalactoside and purified by GST-Bind resin chromatography. The purified fusion protein was applied to immunize New Zealand White rabbits to raise a polyclonal anti-TRIF antiserum (31).

### Immunoblot Assays

The HEK293 cells were washed by using cold PBS and were lysed by using western and IP cell lysis buffer. Protein concentrations of the cell lysates were measured by BCA assay (Pierce) method and equalized with the extraction reagent. Equal amount of the extracts mixed with equal amount of 2× SDS loading buffer and loaded to SDS-PAGE, which subsequently transferred onto PVDF membranes (Millipore) using semi-dry (Bio-Rad Trans Blot Turbo System). Then the membranes were blocked for 90 min at room temperature in 5% dried skimmed milk and were incubated primary antibodies at 4°C overnight. The primary antibodies used in this study were against HA, Flag, Myc, GFP Tag (Santa Cruz, mouse), and GAPDH, β-actin, tubulin (Sigma, mouse), and TRIF (Sigma, rabbit). Then, the membranes were three times with TBST and then incubated with the secondary antibody conjugated with horseradish peroxidase (Beyotime, mouse/rabbit) for 60 min at room temperature. The immunoreactive proteins were detected with WesternBright™ ECL (Advansta), and digital imaging was performed with a cold CCD camera.

### Immunoprecipitation Assay

For immunoprecipitation (IP) experiments, HEK293 cells were seeded onto 10 cm^2^ plate overnight then were co-transfected with 5 µg indicated plasmids. At 48 h post-transfection, the cells were washed three times with ice-cold PBS. Then the cells were lysed with 500 µl western and IP lysis buffer (Beyotime) containing protease inhibitor cocktail (Bitake) at 4°C for 30 min on a rocker platform. Then the cellular debris was removed by centrifugation at 14,000 *g* for 15 min at 4°C. After centrifugation, the supernatant was transferred into a fresh centrifuge tube and incubated with 50 µl protein A + G (Sigma) together with 1 µg monoclonal anti-Flag (Sigma) overnight at 4°C with constant and softly agitation. The following day, the IP protein was collected by centrifugation at 2,500 *g* for 5 min at 4°C. Then beads were washed five times with western and IP lysis buffer and resuspended in 60 µl 2× SDS loading buffer. The immunoprecipitates and whole-cell lysates (WCLs) were analyzed by immunobloting.

### Fluorescent Microscopy

HEK293 cells were seeded onto 24-well plate and transfected by using Lipofectamine™ 2000 (Invitrogen) with indicated plasmids for 48 h. Then the images were obtained under a fluorescence microscope (Leica).

### RNA Interference

The miiuy croaker IRF3-specific siRNA (si-IRF3) and TRIF-specific siRNA (si-TRIF) were 5′-GCUUCAAACUGGUCUCUGATT-3′ (sense), 5′-UCAGAGACCAGUUUGAAGCTT-3′ (antisense), and 5′-GAGACAACUACCUUGCUAGTT-3′ (sense), 5′-CUAGCAAGGUAGUUGUCUCTT-3′ (antisense), respectively. The scrambled control RNA (si-Ctrl) sequences were 5′-UUCUCCGAACGUGUCACGUTT-3′ (sense) and 5′-ACGUGACACGUUCGGAGAATT-3′ (antisense). Miiuy croaker macrophages or HEK293 cells were transfected with 50 nM of each siRNA for up to 36 h before SCRV stimulation using Lipofectamine 3000™.

### Statistical Analysis

All the experiments were performed at least three independent experiments (*n* ≥ 3) with three technical replicates for each experiment. The relative gene expression data was obtained by using the 2^−ΔΔCT^ method, and comparisons between groups were analyzed by one-way analysis of variance followed by Duncan’s multiple comparison tests (33). Results are expressed as mean ± SE, and differences between means were with *p* values of <0.05 considered to be statistically significant.

## Results

### IRF3 Is Upregulated After Poly(I:C) and SCRV Induction

**Figure 1:**
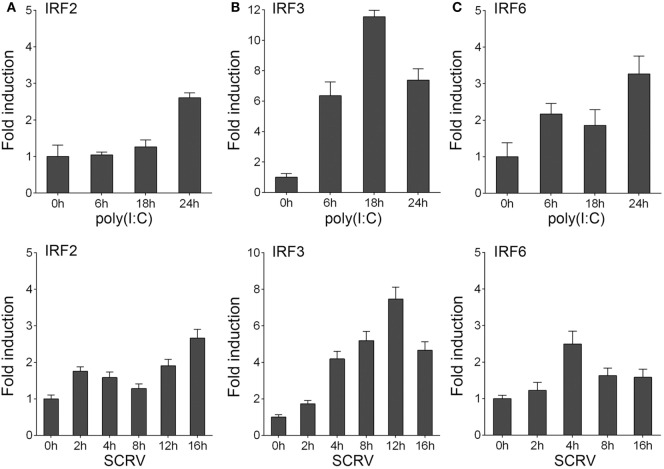
Inducible expression patterns of IRF3. **(A–C)** Miiuy croaker macrophages were seeded onto 12-well plate overnight and stimulated with poly(I:C) (2 µg/ml) for 6, 18, and 24 h, or SCRV (MOI = 5) for 2, 4, 8, 12, and 16 h. Then total RNAs were extracted to examine the expression of IRF2, IRF3, and IRF6 by qRT-PCR. The data were normalized to β-actin. Results are standardized to 1 in control cells.

To determine whether the expression of host IRF3 regulated by viral infection, miiuy croaker macrophages were stimulated with polyriboinosinic polyribocytidylic acid [poly(I:C)] or infected with fish rhabdovirus SCRV. SCRV is a double-stranded RNA virus, which is used as a presumably physiological form of poly(I:C). The expression of IRF3 was then detected by qRT-PCR. The results showed that IRF3 expression was upregulated in both conditions. The results also suggested that IRF3 could be activated to a greater extent after poly(I:C) stimulation and SCRV infection in host cell than after the administration of IRF2 and IRF6 (Figure 1). These data demonstrated the important role of IRF3 in viral infection.

### IRF3 Inhibits the NF-κB Signaling Pathway

**Figure 2:**
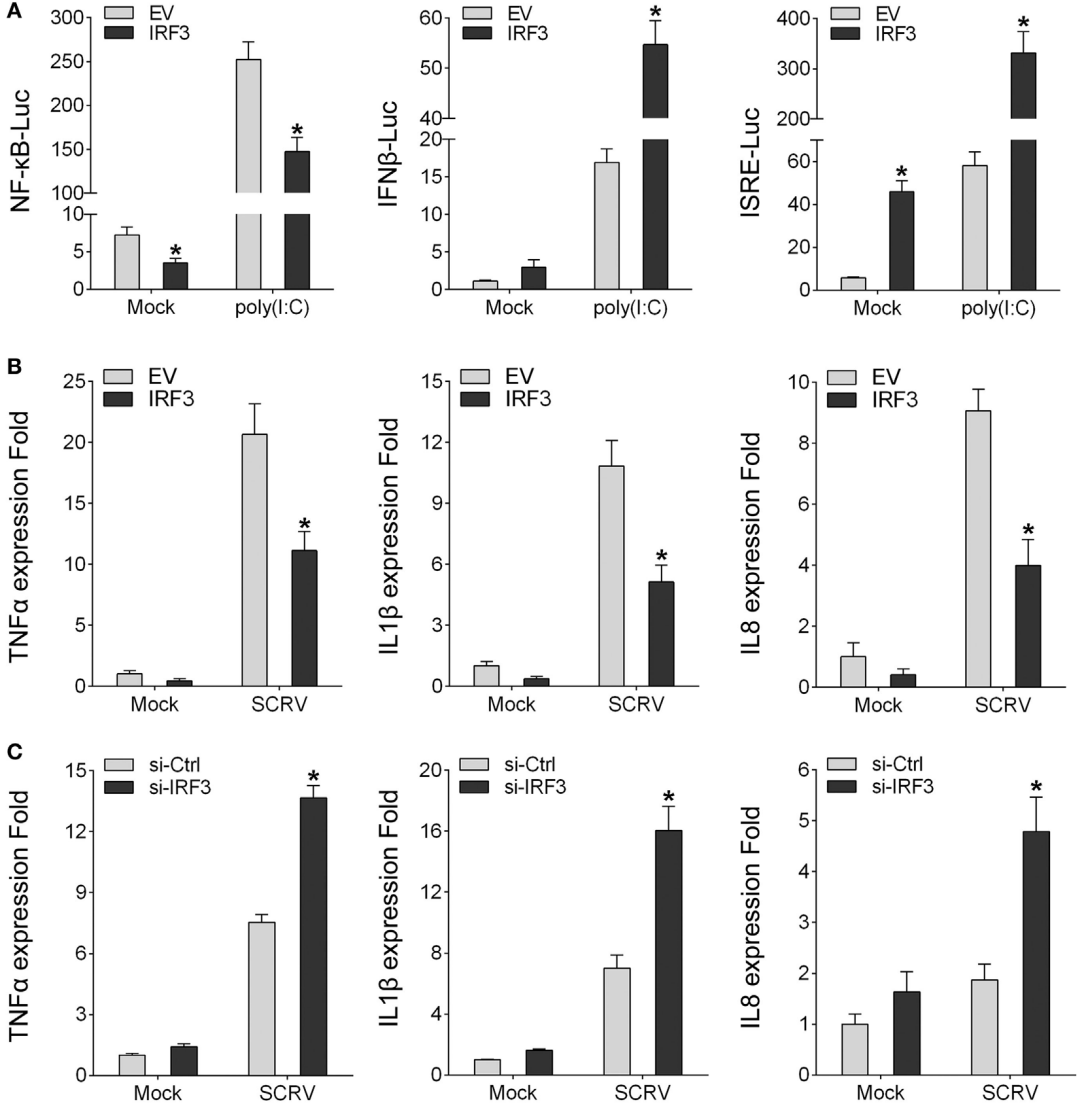
IRF3 can inhibit poly(I:C)-induced NF-κB pathway and expression of downstream cytokines. **(A)** HEK293 cells were co-transfected with empty vector or IRF3 together with NF-κB, IFNβ, or ISRE reporter gene, after 36 h, stimulated with poly(I:C) for 12 h, then the luciferase activity was measured, the luciferase activity value was achieved against the Renilla luciferase activity. **(B)** Miiuy croaker macrophages were transfected with empty vector or IRF3, after 36 h, infected with SCRV (MOI = 5) for 12 h, then total RNAs were extracted to examine the expression of TNFα, IL1β, and IL8 by qRT-PCR. **(C)** Miiuy croaker macrophages were seeded on 24-well plate overnight and transfected with the scrambled control RNA or si-IRF3, after 36 h, infected with SCRV for 12 h, then total RNAs were extracted to examine the expression of TNFα, IL1β, and IL8 by qRT-PCR. *p < 0.05 versus the controls.

To identity the regulatory role of IRF3, IRF3 was transfected into HEK293 cells together with different reporter gene (NF-κB, IFNβ, and ISRE), respectively, then the HEK293 cells were treated with poly(I:C). The result showed that, among the signaling pathways induced by poly(I:C), only NF-κB signaling pathway was inhibited by IRF3 (Figure 2A). To further confirm the role of IRF3, IRF3 plasmids were transfected into miiuy croaker MKC cells and then infected with SCRV. qRT-PCR analysis indicated that IRF3 overexpression inhibited the transcription of downstream cytokines of NF-κB signaling pathway, including TNFα, IL1β, and IL8 (Figure 2B). Furthermore, IRF3-siRNA was transfected into the macrophage and qRT-PCR was performed to assess the expression of TNFα, IL1β, and IL8 cytokines in the IRF3 knockdown cells. SCRV treatment increased TNFα, IL1β, and IL8 expression at 12 h post-infection in the IRF3 knockdown cells as compared with the control cells (Figure 2C). These data suggested that IRF3 might inhibit poly(I:C)-induced NF-κB signaling pathway.

### IRF3 Inhibits the TRIF-Mediated NF-κB Signaling Pathway

**Figure 3:**
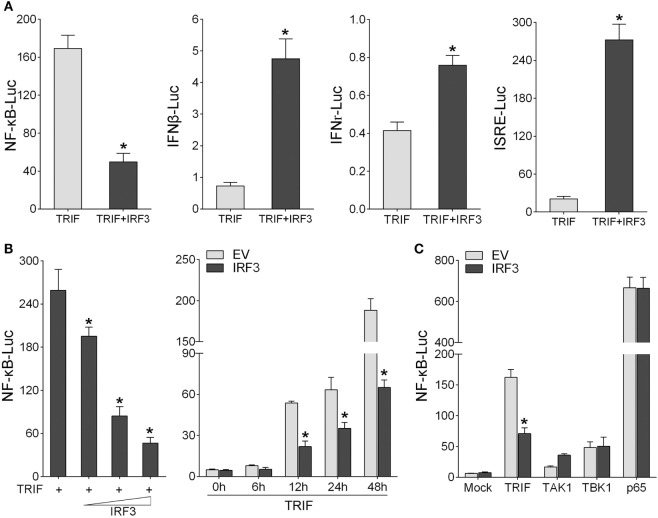
IRF3 represses TRIF-mediated NF-κB signaling pathway. **(A)** HEK293 cells were co-transfected with TRIF and empty vector or IRF3 together with NF-κB, IFNβ, IFNr, or IFN stimulatory response elements reporter gene (ISRE), after 48 h, the luciferase activity was measured. **(B)** The concentration gradient experiment of IRF3 expression plasmid within TRIF and NF-κB reporter gene was conducted (left). After 48 h, the luciferase activity was measured. After co-transfected with TRIF and IRF3 expression plasmids, together with NF-κB reporter gene, the luciferase activity was measured at different time points (right). **(C)** HEK293 cells were co-transfected with NF-κB reporter gene and empty vector or IRF3, together with TRIF, TAK1, TBK1, or p65, after 48 h, the luciferase activity was measured. The luciferase activity value was achieved against the Renilla luciferase activity. *p < 0.05 versus the controls.

To identity the role of IRF3 in NF-κB signaling pathway, first, we determined whether IRF3 can affect the TRIF-mediated NF-κB signaling pathway. According to the results of luciferase reporter assays, TRIF expression sufficiently activated the NF-κB reporter, and the NF-κB activation was suppressed by co-transfected with IRF3 plasmid compared to control. However, co-transfection with TRIF and IRF3 plasmids was unable to inhibit IFNβ, IFNr, and ISRE reporter genes (Figure 3A). To further determine whether IRF3 can negatively regulate TRIF-mediated NF-κB signaling pathway, we performed the IRF3 concentration gradient and different time points experiments with overexpression of TRIF by NF-κB reporter assays (Figure 3B). Overall, these results indicated that a negative regulation effect was intensified at increased amount of IRF3 expression plasmid. In addition, the results showed that IRF3 could not inhibit NF-κB pathway mediated by TRIF downstream genes (Figure 3C). Consequently, IRF3 can inhibit TRIF-mediated NF-κB signaling pathway.

### IRF3 Promotes TRIF Degradation

**Figure 4:**
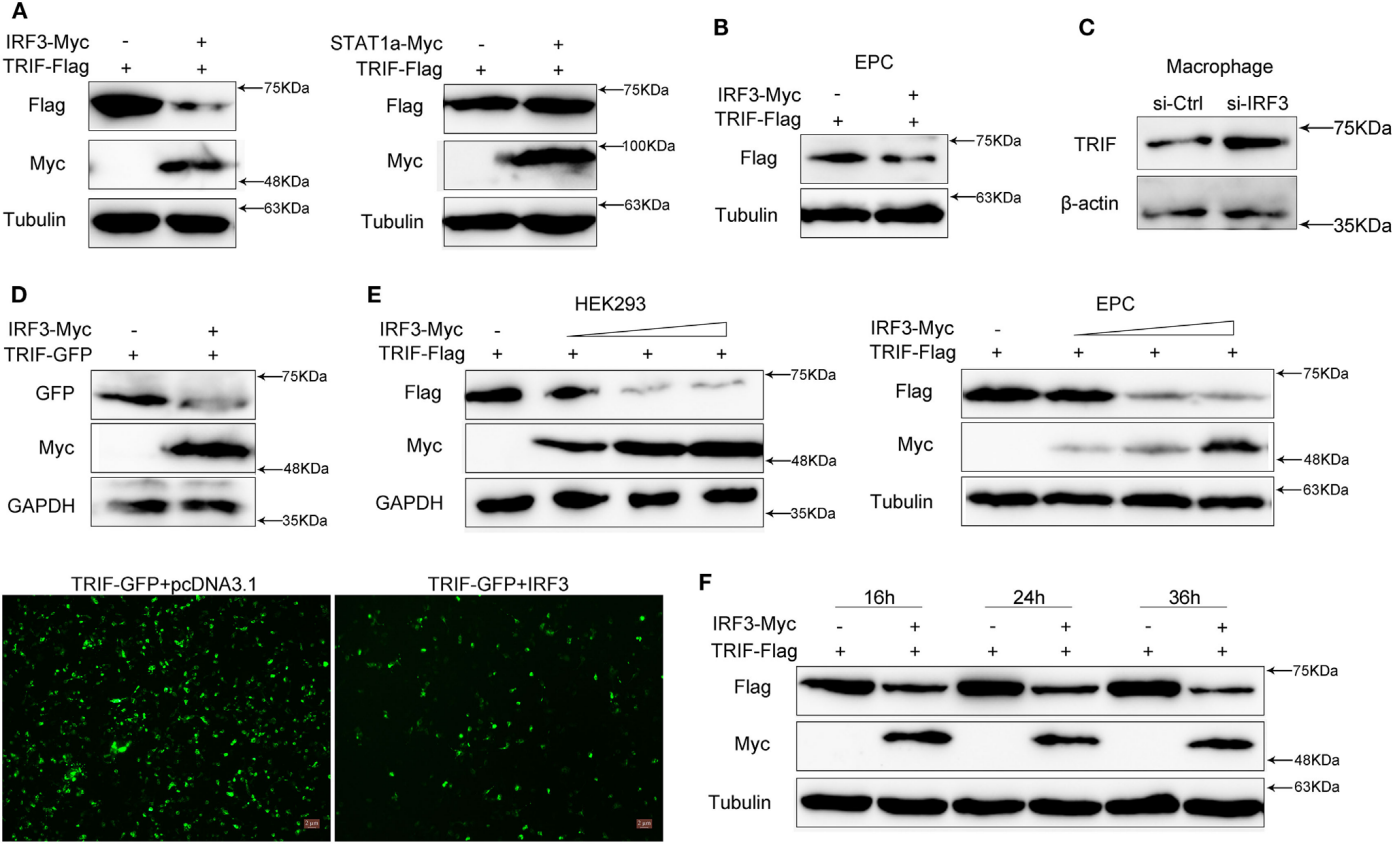
IRF3 promotes the TRIF degradation. **(A)** HEK293 cells were co-transfected with empty vector, IRF3 (left) or STAT1a (right) together with TRIF, after 48 h, TRIF protein levels were determined by immunoblot assays and normalized to tubulin. **(B)** EPC cells were co-transfected with empty vector or IRF3 together with TRIF, after 48 h, TRIF protein levels were determined by immunoblot assays and normalized to tubulin. **(C)** Miiuy croaker macrophages were transfected with the scrambled control RNA (si-Ctrl) or si-IRF3. After 48 h, TRIF protein levels were determined by Western blot and normalized to β-actin. **(D)** HEK293 cells were co-transfected with empty vector, IRF3 together with TRIF-GFP, after 48 h, the fluorescence signals of TRIF-GFP were detected by fluorescence microscopy (below), then TRIF protein levels were determined by immunoblot assays and normalized to GAPDH (top). **(E)** The concentration gradient experiment of IRF3 expression plasmid within TRIF was conducted in HEK293 (left) and EPC cells (right) respectively, TRIF protein levels were determined by immunoblot assays and normalized to GAPDH or tubulin. **(F)** The time gradient experiment of IRF3 expression plasmid within TRIF was conducted, TRIF protein levels were determined by immunoblot assays, and normalized to tubulin.

To investigate the effect of IRF3 on TRIF expression, HEK293 cells were co-transfected with TRIF and IRF3 or negative control STAT1a plasmids, after 48 h transfection, the TRIF expression was examined by immunoblot assays. The results indicated that the protein levels of TRIF were decreased in the presence of IRF3; however, STAT1a had no effect on TRIF protein levels (Figure 4A). To confirm the effect of IRF3 on TRIF expression in fish cells, the EPC cells were co-transfected with TRIF and IRF3 plasmids, after 48 h transfection, the TRIF expression was examined by immunoblot assays. The results indicated that the protein levels of TRIF also decreased in the presence of IRF3 in fish cells (Figure 4B). To further confirm the effect of endogenous IRF3 on TRIF expression in miiuy croaker, the miiuy croaker macrophages were transfected with IRF3-siRNA which was used by inhibiting IRF3 expression. Then the expression of endogenous TRIF protein was examined by immunoblot assays. The results showed that TRIF protein could be upregulated when IRF3-siRNA was transfected (Figure 4C). To further confirmed the role of IRF3 on TRIF expression, HEK293 cells were co-transfected with TRIF-GFP expression plasmid together with IRF3 or the empty vector. As shown in Figure 4D, the fluorescence signals of TRIF-GFP markedly decreased in the presence of IRF3 compared with empty vector. Then the cells were lysed and TRIF protein was examined by immunoblot assays with GFP antibody. Based on the assay results, we speculated that IRF3 may promote TRIF degradation. Accordingly, we conducted a concentration gradient in HEK293 or EPC cells respectively; (Figure 4E) and time point experiments (Figure 4F), and the results demonstrated that IRF3 specifically targeted TRIF for protein degradation.

### IRF3 Affects TRIF Downstream Molecules

**Figure 5:**
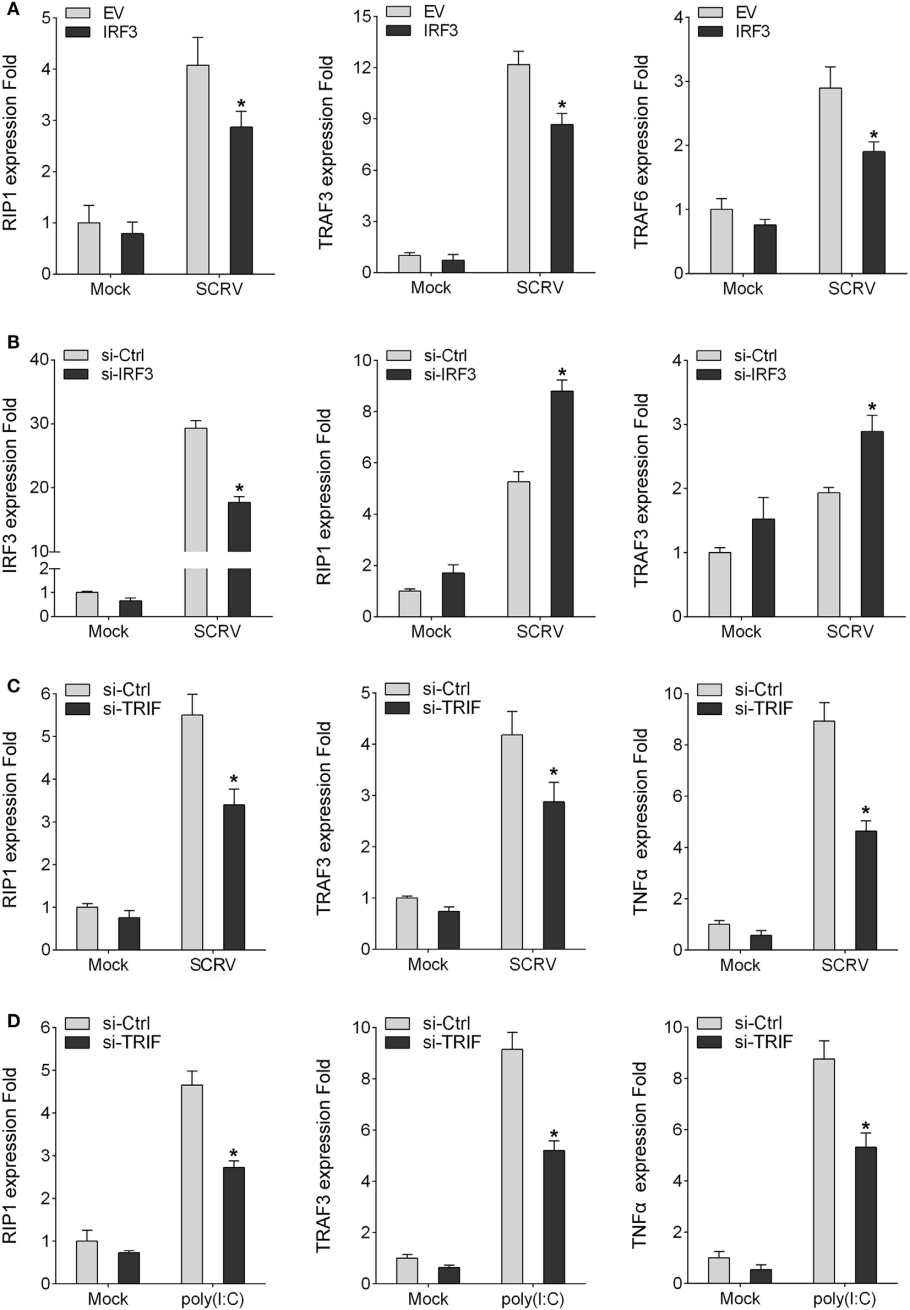
IRF3 affects TRIF downstream molecules. **(A)** MKC were seeded onto 24-well plate overnight and transfected with empty vector or IRF3, after 36 h, infected with SCRV for 12 h, then total RNAs were extracted to examine expression of RIP1, TRAF3, and TRAF6 by qRT-PCR. **(B)** Miiuy croaker macrophages were transfected with the scrambled control RNA (si-Ctrl) or si-IRF3, after 36 h, infected with SCRV for 12 h, then total RNAs were extracted to examine expression of IRF3, RIP1, and TRAF3 by qRT-PCR. **(C,D)** Miiuy croker macrophages were transfected with si-Ctrl or si-TRIF. After 36 h, macrophages were then infected with SCRV for 12 h and the expression of RIP1, TRAF3, and TNFα and stimulated with poly(I:C) for another 12 h and the expression of RIP1, TRAF3, and TNFα were determined. *p < 0.05 versus the controls.

To detect the influence on IRF3 for TRIF downstream molecules, the miiuy croaker MKC cells or macrophages were transfected IRF3 plasmid or IRF3-siRNA respectively, cells were infected SCRV after 36 h transfection, then qRT-PCR was performed. The results showed that the expression of TRIF downstream genes (RIP1, TRAF3, and TRAF6) were downregulated after IRF3 was transfected into the MKC cells (Figure 5A). However, when the cells were transfected with IRF3-siRNA, the expression of RIP1 and TRAF3 were upregulated (Figure 5B). The results indicated that IRF3 can affect the expression of TRIF downstream molecules. The above results indicated that IRF3 promoted TRIF degradation and affected TRIF downstream molecules. To further determine that TRIF protein can be degraded and whether their degradation affected the expression of downstream proteins and cytokines, miiuy croaker-specific TRIF-siRNA was transfected into macrophages. At 36 h post-transfection, the cells were treated with poly(I:C) or infected SCRV for 12 h, then qRT-PCR was subsequently performed. The results showed that the downstream proteins and cytokines of TRIF were downregulated when the cells were transfected with TRIF-siRNA (Figures 5C,D). These dattum indicated that knockdown of TRIF significantly decreased the expression of the downstream proteins and cytokines after poly(I:C) stimulation or SCRV infection in the macrophages.

### Knockdown of IRF3 Potentiates TRIF-Mediated NF-κB Activation

**Figure 6:**
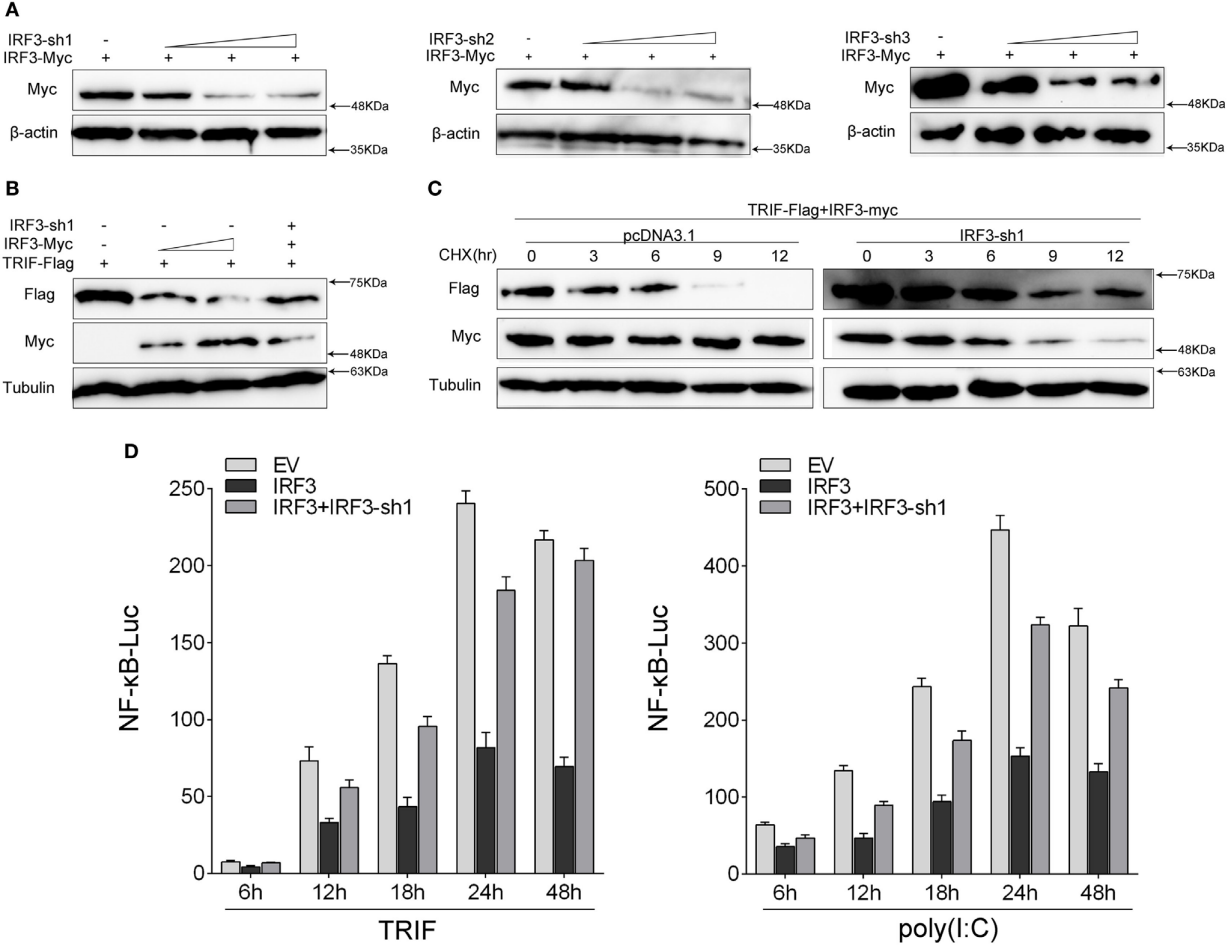
Knockdown of IRF3 relieves the degradation of TRIF and potentiates TLR-mediated NF-κB pathway. **(A)** The IRF3, a different concentration of IRF3-sh1, IRF3-sh2, or IRF3-sh3 plasmids were transfected into HEK293 cells, after 48 h, IRF3 protein levels were determined by immunoblot assays and normalized to β-actin. **(B)** HEK293 cells were co-transfected with TRIF, IRF3, and IRF3-sh1, after 48 h, TRIF protein levels were determined by immunoblot assays and normalized to tubulin. **(C)** HEK293 cells were co-transfected with TRIF, IRF3, and IRF3-sh1 plasmids, at post-transfection 36 h, cells were treated with cycloheximide (100 µg/ml), then cells were lysed at different time point and the expression of TRIF was examined by immunoblot assays. TRIF protein levels were normalized to tubulin. **(D)** HEK293 cells were co-transfected with TRIF, IRF3, IRF3-sh1, together with NF-κB, the luciferase activity was measured at different times (left); after co-transfected with IRF3, IRF3-sh1 plasmid, and NF-κB for 36 h, and then stimulated with poly(I:C) for different times, the luciferase activity was measured (right). The luciferase activity value was achieved against the Renilla luciferase activity. *p < 0.05 versus the controls.

To further confirm the function of IRF3 in the TRIF-mediated NF-κB pathway, we investigated whether knockdown of IRF3 affected TRIF-mediated NF-κB activation. First, we synthesized and constructed three knockdown plasmids designated as IRF3-shRNA1 (IRF3-sh1), IRF3-shRNA2 (IRF3-sh2), and IRF3-shRNA3 (IRF3-sh3), respectively. Then we confirmed that three IRF3-shRNA plasmids efficiently downregulated the expression of IRF3 through immunoblot assays (Figure 6A). HEK293 cells were co-transfected with TRIF, IRF3, and IRF3-shRNA1 plasmids, at post-transfection 48 h, the TRIF expression was then examined by immunoblot assays. The result showed that IRF3-shRNA inhibited IRF3 expression, thereby weakening TRIF degradation (Figure 6B). HEK293 cells were co-transfected with TRIF, IRF3, and IRF3-shRNA1 plasmids, at post-transfection 36 h, and then cells were treated with CHX before cells were lysed. The TRIF protein expression was then examined by immunoblot assays. The results also showed that IRF3-shRNA inhibited IRF3 expression, thereby weakening TRIF degradation (Figure 6C). To demonstrate the inhibitory effect of IRF3 gene on TRIF-mediated NF-κB signaling pathway, IRF3 and IRF3-shRNAs plasmids were co-transfected into HEK293 cells together with TRIF or subsequently subjected to poly(I:C) stimulation after transfection 36 h, and a time gradient experiment was then conducted to confirm the above results, and the data showed that the inhibitory effect of IRF3 was constant at different time points (Figure 6D). Overall, our findings indicate that IRF3 plays a negative regulator role in the TRIF-mediated NF-κB pathway. Furthermore, the negative regulation was weakened when IRF3-shRNA plasmids were co-transfected together with IRF3 and TRIF.

### IRF3 Promotes TRIF Degradation Through Proteasome Pathway

**Figure 7:**
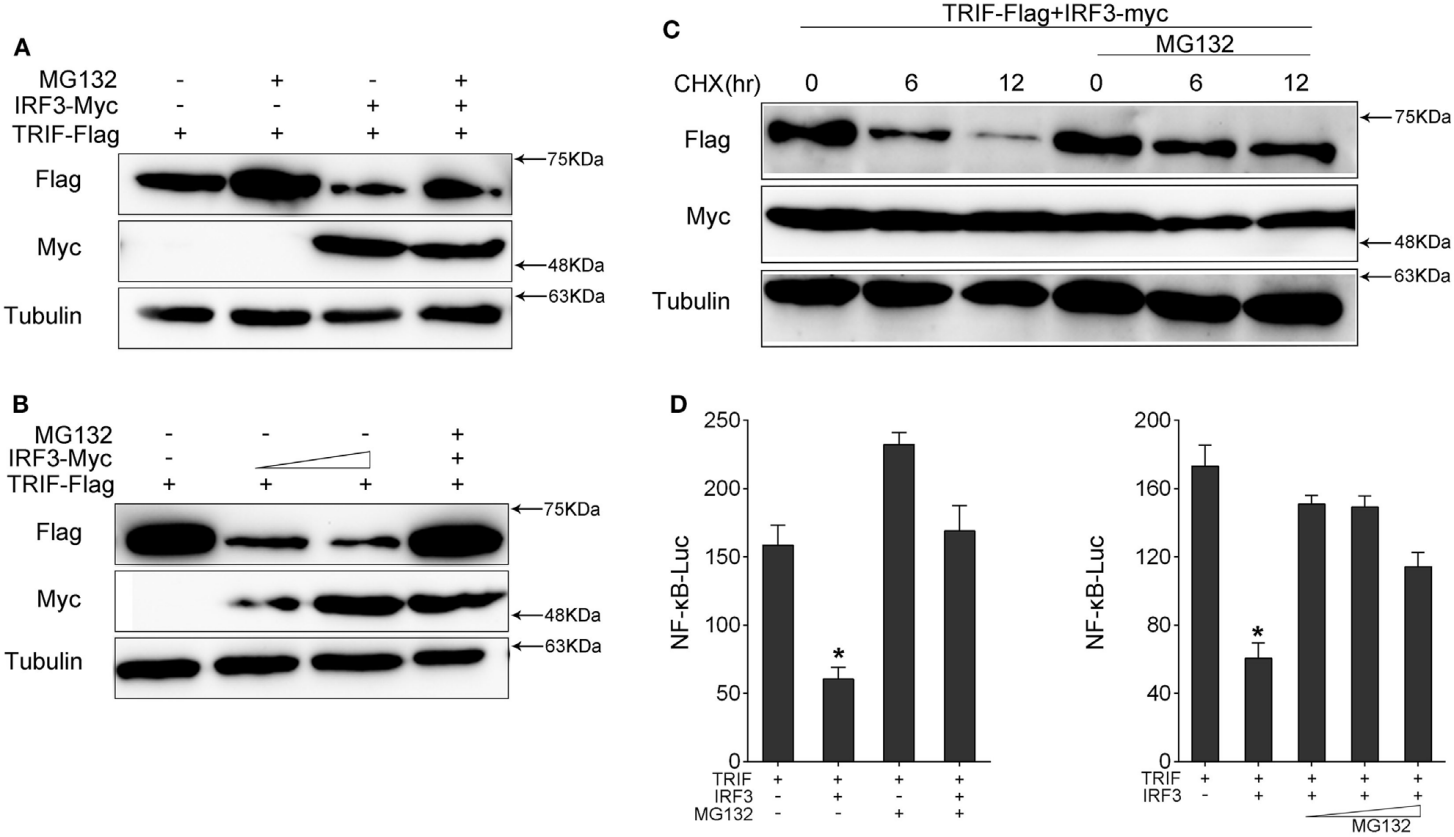
IRF3 induces TRIF degradation through proteasomal pathway. **(A,B)** HEK293 cells were co-transfected with TRIF and IRF3 or concentration gradient of IRF3 plasmids, after 36 h, the cells were treated with DMSO or 10 µM MG132 for 12 h before immunoblot analysis was performed. **(C)** HEK293 cells were transfected with TRIF and IRF3 plasmids, after 36 h, the cells were treated with cycloheximide (100 µg/ml), meanwhile the experiment group treated with DMSO or 10 µM MG132 for different times. TRIF protein levels were determined by immunoblot assays and normalized to tubulin. **(D)** HEK293 cells were co-transfected with TRIF, IRF3, together with NF-κB reporter gene, after 36 h, the cells were treated with DMSO or 10 µM MG132 or concentration gradient MG132 (5, 10, and 15 µM) for 12 h, then the luciferase activity was measured. The luciferase activity value was achieved against the Renilla luciferase activity. *p < 0.05 versus the controls.

To determine which way was used to promote TRIF degradation by IRF3, we co-transfected with TRIF and IRF3 plasmids into HEK293 cells. After 36 h, the protein degradation was prevented by treatment with MG132. The results showed that MG132 blocked TRIF degradation (Figure 7A). TRIF was degradated in a dose-dependent manner with increasing of IRF3, but TRIF protein still can be rescued after MG132 treatment (Figure 7B). Then HEK293 cells were co-transfected with TRIF and IRF3 plasmids, at 36 h post-transfection, the cells were treated with CHX, meanwhile, experimental group cells were treated with MG132 (Figure 7C), the results suggested that IRF3 could promote the TRIF degradation, whereas MG132 could impede the process. Furthermore, HEK293 cells were co-transfected with TRIF and IRF3 plasmids together with NF-κB reporter gene, after 36 h transfection, the cells were treated with MG132 for 12 h before cells were lysed. The activation of NF-κB was subsequently measured by luciferase assays (Figure 7D), and the results indicated that MG132 could relieve the inhibitory effect of IRF3 on TRIF-mediated NF-κB signaling pathway. These results indicated that IRF3 could promote TRIF degradation through proteasome pathway, thereby weakening NF-κB activation.

### Inhibitory Effect of IRF3 Primary Depends on IAD Domain

**Figure 8:**
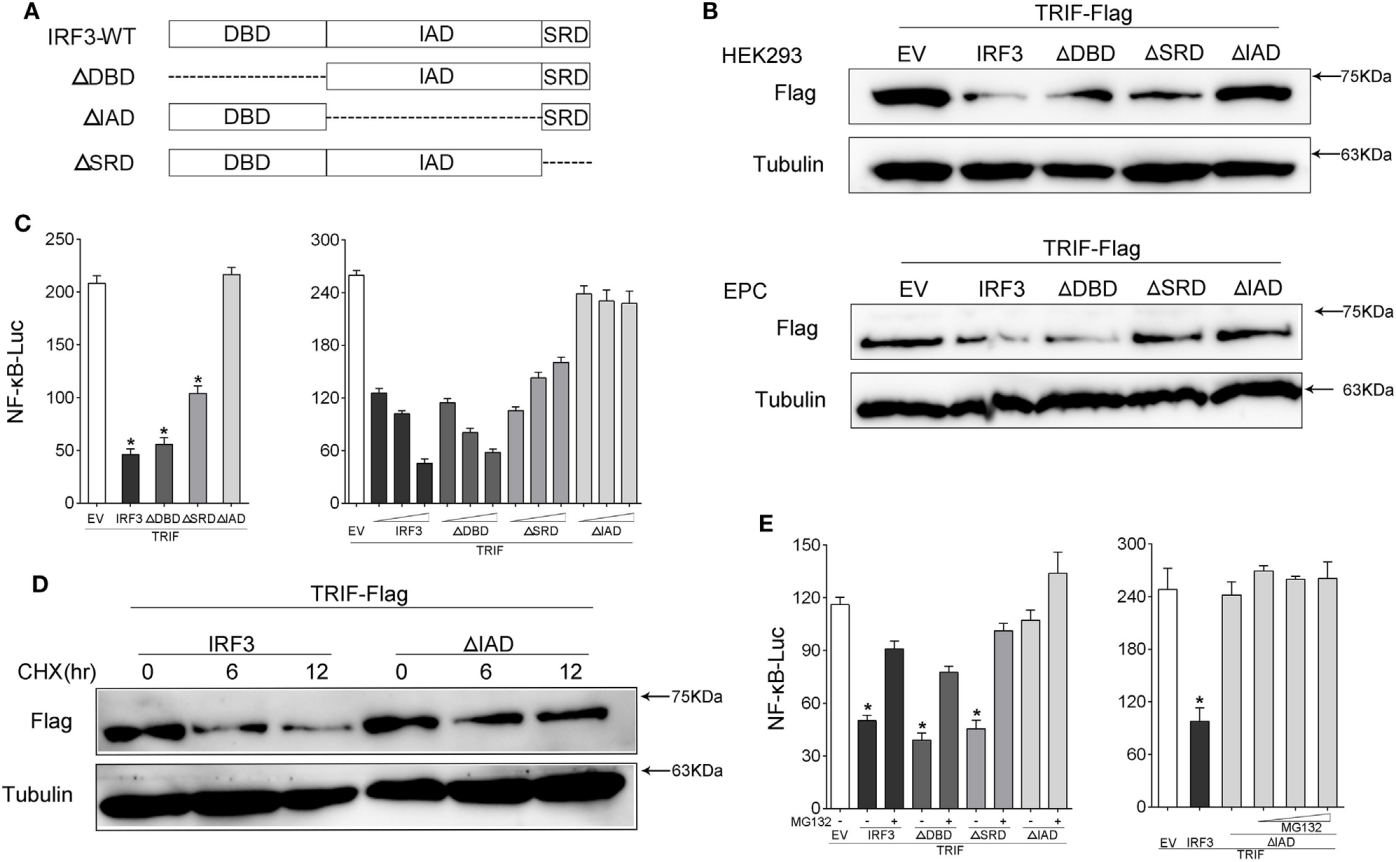
IRF association domain (IAD) of IRF3 is critical for TRIF degradation. **(A)** Schematic diagram of WT and mutants of IRF3 plasmids. **(B)** HEK293 and EPC cells were co-transfected with IRF3, ΔDNA binding domain (DBD), ΔIAD, or ΔSRD, respectively, together with TRIF, after 48 h, TRIF protein levels was determined by immunoblot assays and normalized to tubulin. **(C)** HEK293 cells were co-transfected with TRIF, IRF3, ΔDBD, ΔSRD, ΔIAD, or a different concentration IRF3 and the above mutant’s plasmid, together with NF-κB reporter gene. At 48 h post-transfection, the luciferase activity was measured. **(D)** HEK293 cells were co-transfected with TRIF and IRF3 or ΔIAD plasmids, after 36 h, the cells were treated with cycloheximide (100 µg/ml) for different times, then TRIF protein levels were determined by immunoblot assays and normalized to tubulin. **(E)** HEK293 cells were co-transfected with TRIF, IRF3, ΔDBD, ΔIAD, or ΔSRD respectively, together with NF-κB, after 36 h, the cells were treated with DMSO or 10 µM MG132 or concentration gradient MG132 (5, 10, and 15 μM) for 12 h, then the luciferase activity was measured. The luciferase activity value was achieved against the Renilla luciferase activity. *p < 0.05 versus the controls.

To further confirm which domain of IRF3 gene promoted TRIF degradation, which resulted in the disruption of TRIF-mediated NF-κB activation, we constructed the mutants of IRF3, including IRF3ΔDBD (ΔDBD), IRF3ΔIAD (ΔIAD), and IRF3ΔSRD (ΔSRD) (Figure 8A). HEK293 or EPC cells were co-transfected with IRF3 or other IRF3 mutation plasmids respectively together with TRIF, after 48 h transfection, the expression of TRIF protein was examined by immunoblot assays. The results indicated that IRF3 promoted TRIF degradation, which depended on the IAD domain (Figure 8B). The wild type and mutants of IRF3 and TRIF along with NF-κB reporter gene were co-transfected into cells, the luciferase assay results also demonstrated the IAD domain of IRF3 was the main inhibition domain in the TRIF-mediated NF-κB pathway (Figure 8C), while the deletion of the DBD and SRD domain of IRF3 showed no effect on suppressing TRIF-mediated NF-κB pathway. Additionally, the concentration gradient experiments were conducted to confirm the above results. The data suggested that the deletion IAD domain of IRF3 cannot inhibit TRIF-mediated NF-κB pathway. In addition, HEK293 cells were co-transfected with TRIF and IRF3 or IRF3ΔIAD plasmid respectively, at 36 h post-transfection, the cells were treated with CHX. The results suggested that IRF3 could promote the TRIF degradation, while IRF3ΔIAD cannot promote TRIF degradation (Figure 8D). Furthermore, HEK293 cells were co-transfected with TRIF and IRF3 or mutants of IRF3 plasmids together with NF-κB reporter gene were treated with cells for 12 h with MG132 after 36 h transfection. Then cells were lysed and the activation of NF-κB was measured by luciferase assays. The results also showed that inhibitory effect of IRF3 primary depended on IAD domain (Figure 8E). The above results indicated that IAD domain is primary domain of IRF3 that promoted TRIF degradation and the IAD domain of IRF3 inhibited the TRIF-mediated NF-κB signaling pathway through promoting TRIF degradation.

### IRF3 Leads to Elevation of TRIF Polyubiquitination and Shortens Its Half-Life

**Figure 9:**
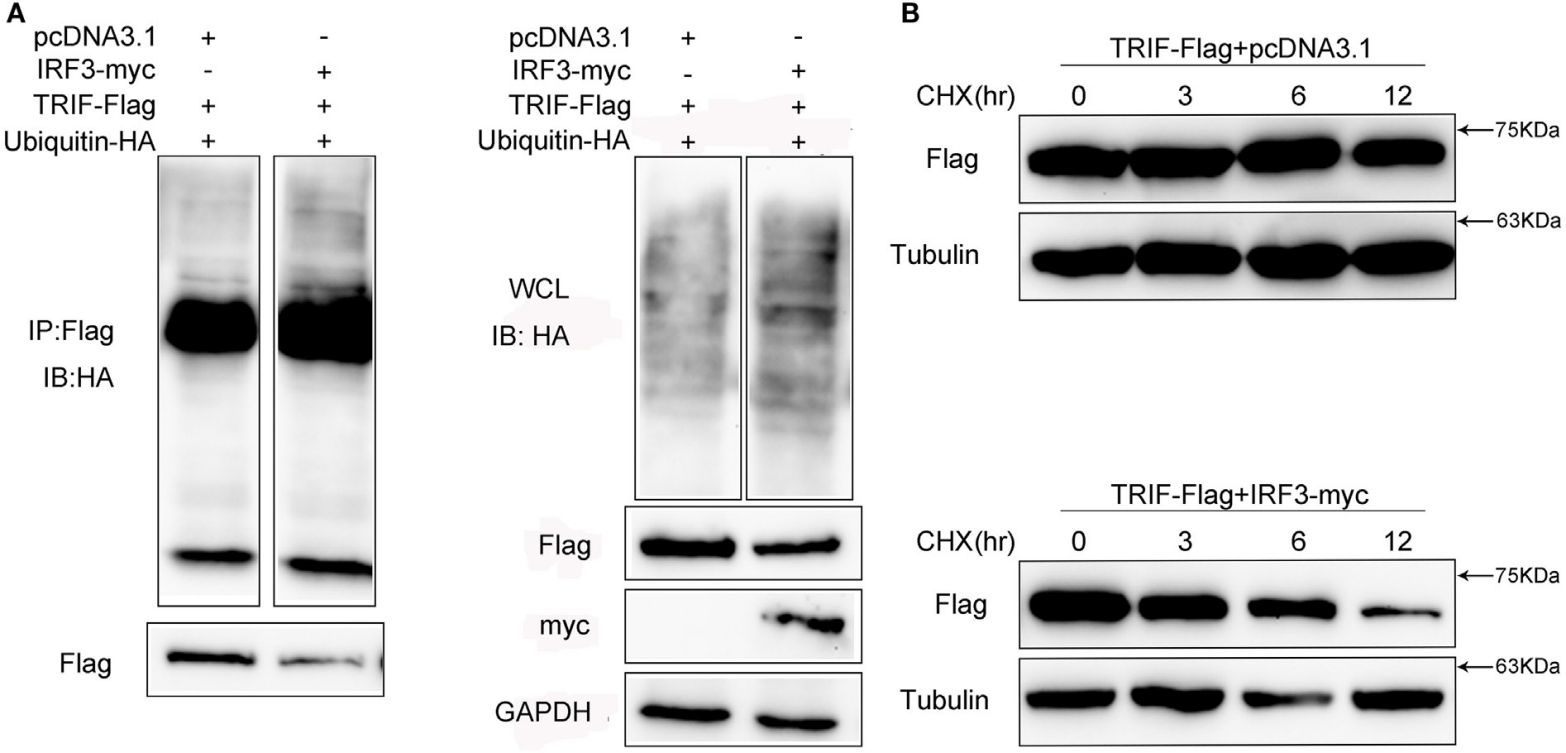
IRF3 leads to TRIF degradation by the ubiquitin–proteasomal pathway. **(A)** HEK293 cells were co-transfected with TRIF, IRF3, and ubiquitin-HA plasmids, after 48 h post-transfection, the cells were lysed and immunoprecipitation analysed with Flag antibody and then WB with antibody against HA. Samples of whole-cell lysate were included as controls. The relative levels of ubiquitinated TRIF are shown below the images after normalization with TRIF level. **(B)** HEK293 cells were transiently co-transfected with TRIF and IRF3 plasmids, after 36 h, the cells were treated with cycloheximide (100 µg/ml) and harvested at different times, followed by immunoblot assays with antibodies against Flag and tubulin. The relative levels of TRIF are shown below the images after normalization with tubulin.

Given that MG132 treatment can restore TRIF protein abundance in cells with IRF3 plasmid, we speculated that the TRIF ubiquitination levels in the cells would increase. To determine whether IRF3 promoted TRIF polyubiquitination, HEK293 cells were co-transfected with ubiquitination plasmid together with TRIF and IRF3 plasmids before the cells were lysed for IP with an antibody against Flag-TRIF. The results of immunoblot assays with HA antibody showed that ubiquitinated TRIF in the cells with IRF3 increased to a greater extent than those in the cells transfected with the empty vector (Figure 9A) and indicated the intensified polyubiquitination of TRIF in the cells with IRF3 expression. Furthermore, the WCL was also detected in the blotting (Figure 9A). The above results indicated that IRF3 induced the elevation of TRIF polyubiquitination in the cells. To test whether the half-life would be shortened, HEK293 cells were co-transfected with TRIF and IRF3 plasmids. The cells were then treated with CHX before they were lysed at different time points. Immunoblotting was subsequently performed. In the presence of IRF3, the TRIF level decreased at a higher rate than those in the cells transfected with the empty vector (Figure 9B). At 3, 6, 9, and 12 h after the CHX treatment, the protein levels of TRIF in the cells with IRF3 expression were degraded rapidly. By contrast, TRIF in the cells transfected with the empty vector were degraded relatively slow (Figure 9B). This result also suggested that IRF3 expression can lead to the elevation of TRIF polyubiquitination.

## Discussion

As one of transcriptional regulators, members the NF-κB/IκB family can promote the expression of over 100 target genes, the majority of which involved in the host immune responses (34). However, excessive inflammation may damage airframe and result in autoimmune diseases. Therefore, to maintain immune balance, the NF-κB signaling pathway is tightly regulated. For instance, WWP2 (23), NLRX1 (6), and TRIM38 (35) can negatively regulate TLR-induced signaling pathway by targeting different gene for degradation. In this study, we found that IRF3 negatively regulates TRIF-mediated NF-κB signaling pathway by targeting TRIF for degradation in fish. Overexpression or knockdown of IRF3 could inhibit TRIF-mediated NF-κB signaling pathway, whereas its knockdown has an opposite effect. Mechanistically, IRF3 promotes TRIF degradation through the ubiquitin–proteasome pathway. However, direct interaction between the IRF3 and TRIF was not observed through co-immunoprecipitation (data not shown). Therefore, our working hypothesis is that the IRF3 would recruit an E3 ligase or enhance its activity and then induced the TRIF ubiquitination. Furthermore, we find that the IAD domain of IRF3 is crucial to proteasomal degradation of TRIF. Therefore, we speculate that IRF3 inhibits TRIF expression, thereby decreasing the expression of downstream cytokines.

In innate and adaptive immune responses, IFN gene expression is regulated by IRF family proteins (36, 37). Currently, 11 members of the IRF family have been found in fish (38). Most IRFs can positively regulate IFN expression (39) and are divided into the positive regulators (IRF1, IRF3, IRF4, IRF5, IRF6, IRF7, IRF8, IRF9, and IRF11) and negative regulators (IRF2 and IRF10) according to the capacity of IFN regulation (40–45). However, in addition to regulating the expression of IFN, increasing evidence suggests that IRFs are also involved in the regulation of many innate signaling pathways. For instances, IRF4 (24) and IRF5 (25) affect downstream of the TLR–MyD88 signaling pathways; and IRF8 interacts with TRAF6 and regulate the production of inflammatory mediators (46). In addition, IRF3 and IRF7 can interact with MyD88 and regulate the IRF-induced IFN response in Atlantic salmon (47). IRF family members have been found and reported that they had different function in different signaling pathway. However, in our study, we first found that IRF3 could negatively regulate the TRIF-mediated NF-κB signaling pathway. This feedback regulation mechanism ensures the relative balance of immunity.

Most IRF proteins contain an N-terminal DBD and a C-terminal IAD. IRF3 has another domain (SRD) at the C-terminus apart from DBD and IAD. The DBD is crucial for interaction of IRF with IFN stimulatory response elements (39, 48). A previous study indicates that the DBD is the core unit that interacts with the promoter of target genes engaged in the IFN pathway. Moreover, except for IRF1 and IRF2, the IADs form a homodimer that recruits other transcription factors (37) and are involved in protein–protein interaction with other IRFs or related molecules (44). In this study, we found that the IAD domain of IRF3 affected TRIF degradation, and the inhibitory effect was weakened, even eliminated when IAD is deleted. This result indeed confirms that the IAD domain of IRFs interacts with related molecules.

Most IRFs found in fish are homologous with those found in mammals, but several differences still exist between fish and mammals. In particular, IRF4a, IRF4b, and IRF10 are found in fish, but not in mammals, suggesting that conservation and evolution coexist from lower vertebrates to mammals. For instances, IRF4 functions as a negative regulator of IFN expression through competing with IRF5 to interact with MyD88 in mammals (24). By contrast, IRF4 promotes the transcription of IFN as a positive regulator in fish (40). Moreover, IRF6 has no effect on IFN production in mammals, while it can significantly activated IFN transcription in fish (40). IRF3 is a typical ISG that is upregulated after stimulation with IFN or a virus. However, in this study, we discovered that IRF3 could negatively regulate TRIF-mediated NF-κB signaling pathway by targeting TRIF for degradation. In addition, to detect whether the similarly role in mammal, we found that IRF3 of human also may promote the degradation of TRIF (date not shown). This result suggests that the function of same gene is conserved from lower vertebrate to mammals.

The current study has identified that IRF3 as a novel negative regulator inhibits TRIF-mediated NF-κB signaling pathway by targeting TRIF for degradation through the ubiquitin–proteasomal degradation pathway in fish. These dattum enrich content of TLR signaling pathway in teleosts, and provide new insights into the regulatory mechanism in mammals.

## Author Contributions

Conceived and designed the experiments: TX. Performed the experiments: XZ, RH, XY, and TX. Analyzed the data: XZ and TX. Wrote the paper: XZ and TX.

## Conflict of Interest Statement

The authors declare that the research was conducted in the absence of any commercial or financial relationships that could be construed as a potential conflict of interest.
